# Supplementary material for: Plants on Rich-Magnesium Dolomite Barrens: A Global Phenomenon
Source: Biology (Basel). 2021 Jan 8;10(1):38. doi: 10.3390/biology10010038 (PMC7826976; doi:10.3390/biology10010038)
Supplement: Supplementary file 1 [file biology-10-00038-s001.pdf]

Table S1. Population genetic parameters estimated for dolomite and reference flora. Abbreviations: H_s = mean within-population gene diversity. Dif. = population differentiation. the following indicators apply: † = G_{st} or ‡ = F_{st}; among-population variance from AMOVA)

Citation	Taxon	Data Type	H_s	Dif. (F_{ST} or G_{ST})
[68]	*Jurinea pinnata*	AFLP	0.135-0.198	0.37‡
[127]	*Convolvulus boissieri*	AFLP	0.033-0.127	0.395‡
[124]	*Viola cazorlensis*	ISSR	0.215	0.104‡
[125]	*Helianthemum apeninus subsp. estevei*	SSR	0.47	
[125]	*Helianthemum panosum*	SSR	0.593	
[129]	*Erigeron parishii*	Alloenzyme	0.16	0.12†
[130]	*Eriogonium ovalifolium var. vineum*	Alloenzyme	0.19	0.07†
[131]	*Astragalus albens*	Alloenzyme	0.139	0.01†
[92]	Gypsum plants		0.196	0.423†. 0.466‡
[117]	Rare			0.212
	Common			0.198
